# White Matter Microstructure and Cognitive Function in Young Women With Polycystic Ovary Syndrome

**DOI:** 10.1210/jc.2015-2318

**Published:** 2015-11-17

**Authors:** D. Aled Rees, Maneesh Udiawar, Rok Berlot, Derek K. Jones, Michael J. O'Sullivan

**Affiliations:** Institute of Molecular and Experimental Medicine (D.A.R., M.U.), School of Medicine and Cardiff University Brain Research Imaging Centre (M.U., D.K.J., M.J.O.), School of Psychology, Cardiff University, Cardiff CF24 4HQ, United Kingdom; Department of Basic and Clinical Neuroscience (R.B., M.J.O.), Institute of Psychiatry Psychology and Neuroscience, King's College London, London SE5 9RX, United Kingdom; and Department of Neurology (R.B.), University Medical Centre Ljubljana, Zaloska cesta 2, 1000 Ljubljana, Slovenia

## Abstract

**Context:**

Polycystic ovary syndrome (PCOS) is a disorder characterized by insulin resistance and hyperandrogenism, which leads to an increased risk of type 2 diabetes in later life. Androgens and insulin signaling affect brain function but little is known about brain structure and function in younger adults with PCOS.

**Objective:**

To establish whether young women with PCOS display altered white matter microstructure and cognitive function.

**Patients, interventions, and main outcome measures:**

Eighteen individuals with PCOS (age, 31 ± 6 y; body mass index [BMI] 30 ± 6 kg/m^2^) and 18 control subjects (age, 31 ± 7 y; BMI, 29 ± 6 kg/m^2^), matched for age, IQ, and BMI, underwent anthropometric and metabolic evaluation, diffusion tensor MRI, a technique especially sensitive to brain white matter structure, and cognitive assessment. Cognitive scores and white matter diffusion metrics were compared between groups. White matter microstructure was evaluated across the whole white matter skeleton using tract-based spatial statistics. Associations with metabolic indices were also evaluated.

**Results:**

PCOS was associated with a widespread reduction in axial diffusivity (diffusion along the main axis of white matter fibers) and increased tissue volume fraction (the proportion of volume filled by white or grey matter rather than cerebrospinal fluid) in the corpus callosum. Cognitive performance was reduced compared with controls (first principal component, *t* = 2.9, *P* = .007), reflecting subtle decrements across a broad range of cognitive tests, despite similar education and premorbid intelligence. In PCOS, there was a reversal of the relationship seen in controls between brain microstructure and both androgens and insulin resistance.

**Conclusions:**

White matter microstructure is altered, and cognitive performance is compromised, in young adults with PCOS. These alterations in brain structure and function are independent of age, education and BMI. If reversible, these changes represent a potential target for treatment.

The precursors of late-life cognitive decline can be traced back to mid adult life. Risk states in middle age include behavioral factors (eg, smoking), aspects of cardiovascular function (eg, hypertension and cardiac structure) and metabolic traits. Metabolic factors shown to confer risk in cohort studies include diabetes and prediabetes: in the Framingham Offspring Study, individuals with prediabetes and diabetes had smaller brain volumes and poorer cognitive performance, especially on tests of executive function and visual memory (1), compared with those with normal glucose regulation. Adiposity, indexed by body mass index (BMI), has also been associated with subsequent dementia (2, 3).

Polycystic ovary syndrome (PCOS) is characterized by a high prevalence of obesity, hyperandrogenism and insulin resistance, leading to an increased risk of type 2 diabetes mellitus. An unfavorable metabolic profile persists after menopausal transition as a result of increased androgen levels and decreased estrogen levels with further exacerbation of insulin resistance, chronic inflammation and adiposity (4). There have been few investigations of the effect of PCOS on the brain. One internet-based study compared neuropsychological functioning in right-handed women with and without PCOS, finding no evidence of an effect of hyperandrogenism or hyperestrogenism on cognitive function (5). One other study described poorer performance on tests of verbal fluency, verbal memory, manual dexterity, and visuospatial working memory in PCOS, but importantly, the comparison group of healthy women was not matched for BMI (6).

Some early evidence suggests that metabolic risk states may have an influence on brain structure. Diffusion tensor magnetic resonance imaging (MRI) is a technique that is sensitive to the structure and organization of white matter in the brain, providing unprecedented insight into the organization of white matter fibers and tracts. Furthermore, it provides quantitative indices of white matter microstructure. Diffusion tensor MRI studies have shown how white matter microstructure evolves in the developing and ageing brain and have also revealed subtle damage in early cognitive decline and in association with vascular risk states. Both type 1 and type 2 diabetes are associated with altered microstructural measures derived from diffusion tensor MRI (7–9). Several studies have now shown associations between BMI and white matter microstructure (10–12). Furthermore, in type 2 diabetes, alterations in tract microstructure have been found to correlate with cognitive performance (7). A common difficulty in interpreting these studies is that metabolic features tend to occur in clustered syndromes, for example, metabolic syndrome, rather than as isolated abnormalities. Consequently, the critical factors that most influence brain structure and function are unclear. This is also true of previous cognitive studies in PCOS that might have been confounded by BMI (6). Defining the main factors that drive end-organ damage in the brain is crucial to the construction of preventative strategies. Carefully controlled studies, that isolate 1 or a small group of factors, have a role that is complementary to large cohort studies in identifying specific features or mechanisms that might be targeted.

The current study investigated brain structure and cognitive function in women in early adulthood with a diagnosis of PCOS. To investigate the effects of metabolic disturbances specific to PCOS, a control group was recruited that was carefully matched for BMI, the main potential confounder, as well as age and premorbid intelligence.

## Materials and Methods

### Participants

Patients with PCOS (n = 18, 16 Caucasian and 2 Afro-Caribbean) were recruited from the endocrine clinic at the University Hospital of Wales. Diagnosis was according to the Rotterdam criteria (13). Congenital adrenal hyperplasia, Cushing's syndrome, androgen-secreting neoplasms, hyperprolactinemia and thyroid disease were excluded by biochemical testing. Patients were between 18 and 45 years of age. Metabolic exclusion criteria were: pregnancy and breastfeeding, hyperlipidemia or use of lipid-lowering agents, diabetes or use of antidiabetic drugs, and use of antiandrogens within 6 months. Neurological exclusion criteria included: previous or current major psychiatric illness, clinical cerebrovascular disease, previous severe head injury, hypertension, and current substance and alcohol abuse.

Healthy volunteers (n = 18, all Caucasian) were recruited as controls with 1 to 1 matching for age and BMI. For each individual patient, a control was identified matched for age (within 2 y) and BMI (within 2 kg/m^2^). Controls needed to have regular menstrual cycles (menses every 27–32 d). Control subjects with signs of hirsutism or with a personal history of diabetes or hypertension, or a family history of PCOS were excluded. Control subjects were recruited by advertisement among students and staff within the School of Medicine and University Hospital of Wales. Their health status was determined by history, physical examination and hormonal evaluation (testosterone, androstenedione, thyroid function, prolactin, and 17-hydroxyprogesterone). The study was approved by Cardiff University (study sponsors), Cardiff and Vale University Health Board and the South East Wales Research Ethics Committee. All subjects gave written, informed consent.

### Anthropometric and biochemical measurements

Height, weight, waist and hip circumference were measured according to our previously published protocol (14). Blood pressure was measured at the brachial artery of the right arm using a validated semiautomated oscillometric device (Omron 705IT; Omron Corp) after 10 minutes of rest in the seated position; an average of 3 readings taken over a 10-minute period was recorded.

Blood samples were collected after an overnight fast. Serum total cholesterol, high density lipoprotein cholesterol and triglycerides were assayed using an Aeroset analyzer (Abbott Diagnostics); low density lipoprotein cholesterol was calculated using Friedewald's formula. Insulin was measured using an immunometric assay specific for human insulin (Invitron), and glucose was measured using the Aeroset chemistry system (Abbott Diagnostics). High sensitivity C-reactive protein (CRP) was assayed by nephelometry (BN II system; Dade Behring), and total testosterone was measured by liquid chromatography-tandem mass spectrometry (Quattro Premier XE triple quadrupole tandem mass spectrometer; Waters Ltd). Androstenedione was measured by immunoassay (Siemens Healthcare). The intra- and interassay coefficients of variation were all less than 9%.

A standard 75-g oral glucose tolerance test was performed in all participants. Glucose and insulin were measured at 0, 30, 60, 90, and 120 minutes. The areas under the curve (AUCs) for insulin and glucose were calculated using the trapezoid method.

### Cognitive testing

A set of cognitive tests was devised based on previous literature on cognitive dysfunction in metabolic disorders (15, 16) and that included tests of episodic memory, attentional, and executive function. Premorbid IQ was estimated with the National Adult Reading Test Revised and current IQ estimated with the Wechsler abbreviated scale of intelligence (WASI). Short-term and working memory were assessed with Digit Span. Episodic memory was assessed by the Free and Cued Selective Reminding test (17) and the Rey Osterrieth Complex figure. Tests sensitive to attention and executive function included Verbal Trails, Digit Symbol Substitution, Stroop Color-Word Association Test, letter and semantic fluency (with letters “FAS” and the category “animals,” respectively). The Beck depression inventory was also administered.

### MRI data acquisition

MRI was performed on a 3T GE HDx MRI system (General Electric). Diffusion images were acquired with a twice-refocused spin-echo echo planar imaging sequence, providing whole oblique axial (parallel to the commissural plane) brain coverage. Data acquisition were peripherally gated to the cardiac cycle. Data were acquired for 60 slices of 2.4 mm in thickness, with a field of view of 23 cm and an acquisition matrix of 96 × 96. Echo time was 87 milliseconds and parallel imaging (array spatial sensitivity encoding factor = 2) was used. The b-value was 1200 s/mm^2^. In each imaging session, data were acquired with diffusion encoded along 30 isotropically distributed directions and 3 nondiffusion-weighted scans, according to an optimized gradient vector scheme (18). Fluid attenuated inversion recovery scans were also obtained. Acquisition time was 19 minutes.

Images were visually inspected for the presence of white matter hyperintensities. On this basis, 2 subjects were excluded from further analysis.

### Diffusion metrics and tract-based spatial statistics (TBSS)

Diffusion-weighted images were corrected for eddy current and motion distortion using the ExploreDTI software package (19). Diffusion tensor images were then corrected to remove the effects of cerebrospinal fluid contamination using the Free Water Elimination approach (20). Maps of the next microstructural measures were then created. Fractional anisotropy (FA) is a measure of the directionality of diffusion, commonly used in diffusion MRI research studies. FA is high in white matter, especially in major tracts in which axons are packed in a coherent, parallel fashion. It is also increased by the presence of axonal membranes or myelin sheaths that hinder diffusion. Disruption of this coherent organization or loss of axons or myelin generally reduce FA and increase mean diffusivity (MD), a measure of diffusion averaged in all spatial directions. Axial diffusivity (AD) is a measure of diffusion taken along the principal axis of diffusion (usually assumed to be the orientation of fibers in a white matter tract). Radial diffusivity (RD) is a measure of diffusion perpendicular to this principal axis. Some have argued that AD is more sensitive to axonal factors and RD more sensitive to myelination but their precise biophysical correlates are not known. Tissue volume fraction (*f*) represents the proportion of a voxel that is composed of gray or white matter (as opposed to cerebrospinal fluid). One simplified interpretation of this measure would be to view it as a measure of atrophy at a small (microstructural) scale. All measures were calculated as previously described (21).

The above steps produced diffusion measurements at each voxel (imaging element) in the brain. To compare measurements across the PCOS and control groups, these images were all mapped to a single template. This allows measures to be compared voxel by voxel (described as “voxel-based analysis”), with the problem of comparisons across many voxels addressed by one of a number of statistical approaches that are widely accepted in neuroimaging research. The advantage of this approach is that it is free of a priori bias about the likely location of structural alteration in the brain. TBSS is a widely accepted approach for statistical analysis of diffusion data and was implemented in this study. TBSS is part of the FMRIB Software Library image analysis software (Centre for Functional MRI of the Brain, University of Oxford, http://www.fmrib.ox.ac.uk/fsl/, version 5.0) (22). Using TBSS, microstructural measures from the presumed tract centers of each participant were estimated. First, the recommended FMRIB58_FA standard-space image was used as the registration target for each subject's FA maps. The aligned FA data were then projected to the mean FA skeleton. Voxel projections defined in this way were applied to project voxel values of MD, AD, RD, and *f* to the white matter skeleton for each subject. Resulting skeletonized data were used to generate voxelwise statistical analyses.

### Statistical analysis

Principal components analysis was used to generate an unbiased composite measure of cognitive scores. Principal components were derived from the next tests: Digit Span, Free and Cued Selective Reminding test, Rey Osterrieth Complex figure test, Verbal Trails, Digit Symbol Substitution and the letter and semantic fluency test. The first principal component, which accounted for 35% of the variance across all test scores, was used as a summary score and compared across the 2 groups using an unpaired *t* test. A threshold of significance of 0.05 was used for this single comparison. Subsequent comparisons for individual test scores were performed. These were exploratory, so only uncorrected *P* values are provided.

For TBSS, 2-sample unpaired *t* tests for reduced and increased diffusion measures in PCOS patients compared with controls were performed. Additionally, microstructural measures were correlated with the value of the first principal component and hormonal levels in each group separately. Statistical inference was based on nonparametric permutation: 5000 permutations were performed using randomize software (23). Resulting statistical maps were thresholded for *P* < .05, corrected for familywise error, by implementing threshold-free cluster enhancement (24).

In the absence of any previous data in subjects with PCOS, we based our sample size calculations on data from a previous study, which examined the influence of BMI on corpus callosum microstructure (10). Anticipating a broadly similar effect size, we calculated that a sample of 18 participants per group would provide at least 95% power to detect a between-group difference in FA of 0.05 at the 5% significance level.

## Results

### Group differences between PCOS and control participants

Participants in the PCOS group included those with full-blown (n = 8, 44%), non-polycystic ovary (n = 1, 6%), nonhyperandrogenic (n = 5, 28%), and ovulatory (n = 4, 22%) subphenotypes described by the Rotterdam criteria. No individual in either group had evidence of depression based on Beck depression inventory score (scores all <15). The groups were well matched for premorbid intelligence. Table 1 shows demographic, clinical, anthropometric, and metabolic characteristics of the 2 groups. The groups were closely matched for age, BMI, and anthropometric measures. As expected, individuals with PCOS had raised testosterone and androstenedione levels compared with controls. Insulin response to oral glucose challenge (insulin AUC) was higher in those with PCOS, confirming insulin resistance in this group. Two subjects in each group were taking a combined oral contraceptive pill, and 2 in each group were smokers. Five subjects in the PCOS group, and 2 controls had prediabetes (American Diabetes Association criteria), whereas 3 subjects with PCOS and 4 controls had metabolic syndrome according to the International Diabetes Federation definition.

**Table 1.** Anthropometric and Metabolic Characteristics of the Study Population

	PCOS (n = 18, Mean ± SD)	Controls (n = 18, Mean ± SD)	*P* Value
Age (y)	31 ± 6	31 ± 7	.9
Estimated premorbid intelligence	122 ± 4	121 ± 8	.35
Weight (kg)	78 ± 21	76 ± 15	.68
BMI (kg/m^2^)	30 ± 6	29 ± 6	.61
Systolic BP (mm Hg)	119 ± 8	120 ± 11	.96
Diastolic BP (mm Hg)	66 ± 8	69 ± 10	.36
Waist (cm)	91 ± 15	86 ± 13	.31
Hip (cm)	111 ± 16	106 ± 12	.24
Visceral fat area (cm^2^)	31 ± 23	26 ± 14	.46
Subcutaneous fat area (cm^2^)	287 ± 119	298 ± 114	.78
Total fat area (cm^2^)	318 ± 133	324 ± 124	.89
Testosterone (nmol/L)	1.6 ± 0.6	0.9 ± 0.6	.01
Androstenedione (nmol/L)	13.4 ± 7.3	6.8 ± 2.5	.001
hsCRP (mg/L)	1.2 (0.2 ± 21.8)	0.9 (0.1 ± 16.7)	.73
Total cholesterol (mmol/L)	4.6 ± 1.3	4.8 ± 1.1	.67
Triglycerides (mmol/L)	1.2 ± 1.4	1.0 ± 0.5	.52
LDL cholesterol (mmol/L)	2.4 ± 1.4	2.5 ± 1.3	.79
HDL cholesterol (mmol/L)	1.2 ± 0.5	1.3 ± 0.6	.65
HbA1c (mmol/mol)	34.6 ± 2.9	33.6 ± 4.4	.40
Insulin AUC (pmol min/L)	93 151 ± 42 694	61 933 ± 29 614	.04
Glucose AUC (mmol min/L)	764 ± 217	692 ± 133	.24

Estimation of premorbid intelligence was based on the National Adult Reading Test–Revised. hsCRP, high sensitivity CRP; LDL, low density lipoprotein; HDL, high density lipoprotein; AUC, AUC during oral glucose tolerance test.

Cognitive performance was degraded in patients with PCOS compared with controls. For the summary score, the between group difference was significant: *t* = 2.88, *P* = .007. Table 2 summarizes performance on underlying individual cognitive measures.

**Table 2.** Performance on Individual Cognitive Tests

	PCOS (n = 18, Mean ± SD)	Controls (n = 18, Mean ± SD)	*P* Value
Intelligence and executive function			
Digit span forward	12 ± 1.9	13.6 ± 2.2	.02
Digit span backward	7.4 ± 2.5	9.1 ± 2.3	.04
Digit symbol	83 ± 15.8	90.1 ± 12.5	.14
Verbal trails (switching cost)	44.5 ± 21.2	33.4 ± 9.8	.03
Verbal trails (errors)	1.1 ± 1.5	.55 ± 1.1	.23
Letter fluency	38.1 ± 11	39.1 ± 11	.79
Category fluency	39.6 ± 7.5	44 ± 6.3	.06
Stroop Colour Word test (duration)	118.3 ± 22.7	106.4 ± 20.5	.11
WASI (vocabulary)	64 ± 9.2	69.88 ± 5.5	.02
WASI (block design)	49.1 ± 13.7	56.2 ± 8.9	.07
WASI (similarities)	39.5 ± 4.8	43.61 ± 2.8	.04
WASI (matrix reasoning)	27.1 ± 6.6	27.8 ± 3.2	.7
Episodic memory			
FCSRT immediate recall	13.7 ± 1.9	15.4 ± 2.4	.03
FCSRT free recall	35.7 ± 8.9	41.1 ± 8.1	.06
FCSRT total recall	41.7 ± 7.7	44.7 ± 6.1	.20
FCSRT delayed free recall	13.2 ± 2.4	14.9 ± 1.2	.01
FCSRT delayed total recall	15.8 ± 0.4	15.9 ± 0.2	.65
Rey Osterrieth Complex figure	26.7 ± 6.9	30.6 ± 4	.05

FCSRT, Free and Cued Selective Reminding test.

**Figure 1.**
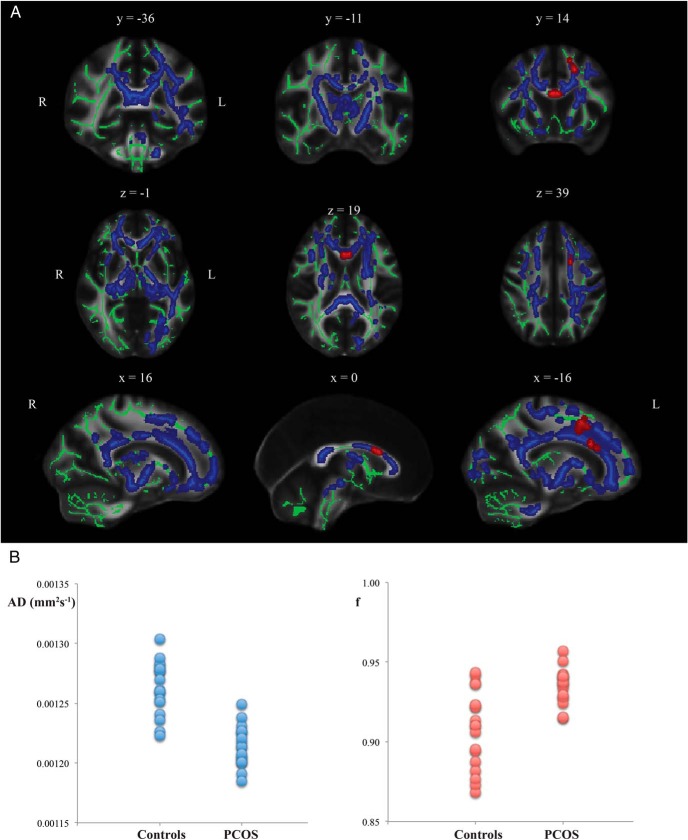
Group differences based on TBSS. A, Mean white matter skeleton voxels showing significantly lower value of AD (blue) and higher value of tissue volume fraction in PCOS (red) compared with healthy volunteers. Displayed results are corrected for familywise error and thresholded for *P* < .05. The white matter skeleton is shown in green. B, Mean values of AD and tissue volume *f* across parts of the skeleton showing a significant group difference for each participant.

Analysis of diffusion MRI images revealed differences in white matter microstructure between PCOS and control groups. Areas of decreased AD in PCOS were found throughout the mean white matter skeleton (Figure 1, blue). In addition, tissue volume fraction was increased in the rostral body of the corpus callosum and parts of anterior white matter (Figure 1, red) in PCOS. There were no significant differences in voxelwise values of FA, MD, or RD between the groups.

### Insulin resistance and white matter microstructure

**Figure 2.**
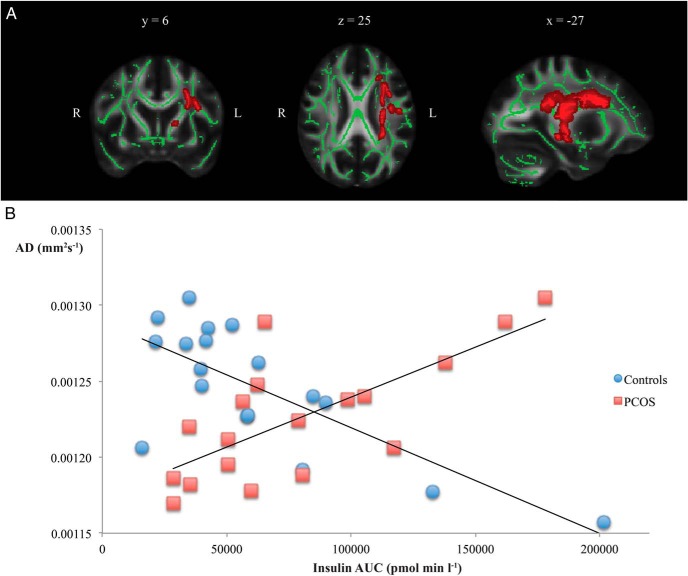
Contrasting associations between white matter microstructure and insulin resistance in PCOS and healthy volunteers. A, White matter skeleton voxels exhibiting a significant group by insulin AUC interaction are shown in red (*P* < .05 corrected for familywise error). B, Mean AD values extracted from the region of significant interaction for each participant and plotted against insulin AUC. The direction of association is positive in PCOS and negative in controls.

Voxelwise correlations between insulin AUC and AD were not significant in either PCOS or controls. However, there was a significant group by insulin AUC interaction (*P* < .05 corrected with familywise error) (Figure 2A), suggesting that the relationship between insulin resistance and white matter microstructure differed in PCOS and control groups. To explore this further, mean values of AD were extracted from this region and plotted against insulin AUC (Figure 2B). Increasing insulin resistance was associated with reduction of AD in controls (Pearson's *r* = −0.75) but with increasing AD in those with PCOS (Pearson's *r* = 0.73), a reversal of the relationship found in controls. The reduction in AD found, on average, in patients with PCOS compared with controls was evident in those with relatively normal insulin AUC (<100 000 pmol min/L) but AD was increased in a subset of PCOS patients with marked insulin resistance.

### Androgens and white matter microstructure

**Figure 3.**
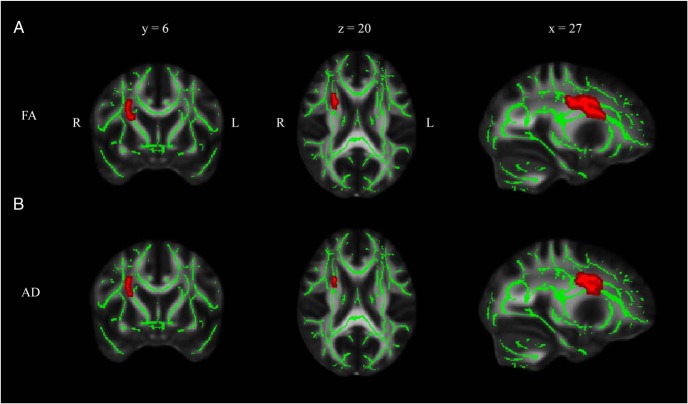
Correlation of testosterone level with microstructural measures in PCOS. White matter skeleton voxels exhibiting a positive correlation with FA (A) and AD (B) are shown in red. Displayed results are corrected for familywise error and thresholded for *P* < .05.

Serum testosterone correlated positively with both AD and FA in the PCOS group (Figure 3). No association was observed in control participants. The direction of effect was consistent with that found between insulin AUC and diffusion metrics, ie, increasing values of AD were associated with both increases in testosterone and insulin AUC (Figure 2B) in patients with PCOS.

### Metabolic status, white matter microstructure, and cognition in PCOS

We found no correlation between cognitive performance and testosterone or insulin AUC in PCOS (testosterone: *r* = −0.12, *P* = .64; insulin AUC: *r* = 0.01, *P* = .98). No significant correlations were observed between microstructural measures and cognition. Similarly, no significant associations with cognitive performance were found in the control group.

## Discussion

PCOS was associated with both subtle decrements of cognitive function and alterations in microstructure of brain white matter. These differences are not attributable to BMI, which was closely matched between groups, as was general intelligence. PCOS is one of the commonest metabolic syndromes, affecting up to 10% of women of reproductive age (25). The public health importance of PCOS is amplified by the association with other rapidly growing public health problems, notably type 2 diabetes and obesity (26). Traditionally, treatment has focused on managing infertility and reducing cardiovascular risk. The current results suggest that the potential consequences of PCOS are wider: the possibility that PCOS represents a midlife risk state for cognitive decline in old age should be explored. The case-control design effectively isolated 2 features of PCOS, insulin resistance and hyperandrogenemia, as possible causes of the alterations in white matter structure and cognition that were observed. Attention to these specific factors would help to define such a risk state and potential treatments more precisely.

The current findings suggest a subtle but widespread erosion of cognitive performance. In an internet-based computerized study, Barnard et al hypothesized that women with PCOS would display enhanced cognitive performance on sexually dimorphic tasks (5). However, no difference was found in performance of mental rotation or spatial location tasks. Contrary to expectation, women with PCOS showed impaired performance on reaction time and word recognition tasks. Schattmann and Sherwin similarly found no differences on tests of mental rotation, spatial visualization, or spatial perception in PCOS (6). However, subjects with PCOS performed less well on tests of verbal fluency, verbal memory, manual dexterity, and visuospatial working memory. Previous studies have often been limited by poor case-control matching. For example, Barry et al found evidence of better visuospatial task performance in women with PCOS compared with subfertile controls, but this difference was no longer significant when age and BMI were controlled for in the analysis (27).

In parallel with metabolic and cognitive differences, microstructural alterations in cerebral white matter were observed in the PCOS group. AD was reduced in a large portion of the white matter skeleton in PCOS and tissue volume fraction was increased in the anterior corpus callosum. Intriguingly, similar findings have been reported in studies on sexual dimorphism. For example, Kumar et al (28) found widespread reductions of axial and RD values in the male brain. The corpus callosum is generally larger in males. Further, a study with myelin-water fraction imaging has illustrated increased myelin density in males in the rostral body and posterior midbody of the corpus callosum (29), possibly concordant with our findings of increased *f* in the anterior part of the corpus callosum even though these studies address structural characteristics that are not necessarily closely correlated. One interpretation of the findings in the present study, therefore, is that an influence of androgens on brain structure produces alterations in PCOS that parallel the characteristics of the male brain.

The finding of a positive association between androgens and AD within the PCOS group was a surprise, given evidence that white matter AD is reduced in PCOS. Hyperandrogenemia and insulin resistance in the PCOS group were associated with increasing AD, despite a mean reduction in the PCOS group as a whole. The likely explanation was a reversal of the normal relationship between microstructure and metabolic status in patients with PCOS, reflected in a significant group by insulin AUC interaction. These findings raise the possibility that the brain responds differently to the effect of androgens in PCOS. Alternatively, there may be significant heterogeneity among individuals that is currently not captured by the coarse diagnostic categories in current use. For example, there may be distinct subtypes of PCOS with and without insulin resistance that differ in a variety of ways that are not yet fully understood; similarly, the presence of increased insulin resistance in some BMI-matched controls suggests heterogeneity in the healthy population.

Previous studies have reported differences in diffusion metrics in both type 2 diabetes (7) and obesity. Type 2 diabetes is associated with increases in AD in a number of specific tracts. Studies in obesity are less consistent, with at least 1 study reporting effects that vary by anatomical location in the brain and include both increases and decreases of AD in different regions (12, 30, 31). These reports illustrate that there is no simple relationship between diffusion measures and better or worse white matter “integrity” (32). The histological basis of alterations in disease states remains unclear and further work is required to aid the interpretation of direction as well as the magnitude of effects. Previous epidemiological studies suggest a complex relationship between BMI and dementia risk. Both high and low body mass states have been associated with future dementia risk, with evidence that the relationship might differ in mid and later life (2). One recent report highlighted this complexity by showing a relationship between low body weight in midlife and dementia (33) contradicting previously held views about midlife obesity (2, 3). The average age of individuals at entry to this recent retrospective study was 55; even less is known about the risk implications of BMI in the twenties and thirties, the age range relevant to the current study.

There were some limitations to the experimental approach. Firstly, we selected our PCOS subjects by the Rotterdam criteria, which embrace a less severe metabolic phenotype than other definitions of the syndrome. However, if anything this might be expected to underestimate the extent of white matter alteration in PCOS subjects with more severe hyperandrogenism and insulin resistance. The Rotterdam criteria also encompass a heterogeneous group, hence future studies should compare distinct PCOS phenotypes, including lean subjects alone, in order to establish whether cognitive function and white matter microstructure are altered in all PCOS patients or only in some. Secondly, we did not capture information on physical activity levels in our study; future studies should look to record this in view of the possible relationship between sedentary behavior and cognitive decline. Thirdly, no clear correlation was found between cognition and white matter structure of relevant regions or connections. This related partly to power. Another factor was the unexpected interaction between group and metabolic factors in their influence on white matter structure.

A key question that follows from these observations is whether alterations in brain structure and function can be reversed, reducing the risk of future cognitive decline. Interestingly, 1 study in PCOS suggested improvement in a single cognitive measure (verbal fluency), after combined treatment with an antiandrogen plus estrogen (34). Based on the current results, both insulin resistance and hyperandrogenism are potential targets, and advanced MRI has a potential role as a biomarker of treatment effect.
